# Human Chorionic Gonadotrophin (hCG) induces changes in IFN-pathway and Interferon-Stimulated Genes (ISGs) on the bovine endometrium at Day 18 of pregnancy

**DOI:** 10.1590/1984-3143-AR2023-0130

**Published:** 2024-03-29

**Authors:** Manuela Wolker Manta, Eduardo Pradebon da Silva, Suzana Rossato Feltrin, Amanda Luiza Prante, Karine de Vargas Aires, Leonardo Guedes de Andrade, Ana Paula da Silva, Carolina dos Santos Amaral, Letícia Minussi Wink, Valério Marques Portela, Alfredo Quites Antoniazzi

**Affiliations:** 1 Universidade Federal de Santa Maria - UFSM, Santa Maria, RS, Brasil; 2 Programa de Pós-graduação em Medicina Veterinária, Universidade Federal de Santa Maria - UFSM, Santa Maria, RS, Brasil

**Keywords:** IFNT, hCG, pregnancy, IFN-pathway, ISGs

## Abstract

We hypothesized that the hCG modulates the expression of IFNT-pathway and ISGs in bovine endometrium during early pregnancy. The aim of the current study is to evaluate the effect of hCG on IFNT-pathway signals and ISGs expression in endometrial cells. For this, 29 non-lactating cross-bread cows were used in the study and submitted to a 9-day fixed-time artificial insemination (FTAI) protocol. The day of the AI was considered Day 0 (D0), and five days (D5) after the FTAI, the cows were allocated into two groups: Control and hCG group, when a hCG group received a single dose of 2.500UI of hCG. On day 18 after FTAI (D18) cows were slaughtered and endometrial tissue samples were collected. There was no difference between the embryo recovery rate of the cows in C compared to the hCG. The hCG group increased the accessory corpus luteum formation rate. The hCG resulted in greater serum progesterone concentration in the hCG group compared to the C on Day 14. Only the expression of IFNAR2 and STAT1 were upregulated on pregnant cows of the hCG group compared to the C group. The pathway genes (JAK1, STAT2, and IRF9) were not regulated. The mRNA abundance of ISG15, MX1, MX2, and OAS1 was upregulated in pregnant cows for hCG group, compared to C group. The results show that the administration of hCG, 5 days after AI, in addition to increasing the serum progesterone, modulates the expression of IFNT-pathway and ISGs on bovine endometrium on Day 18 of pregnancy.

## Introduction

Pregnancy is a highly complex biological process and requires embryo-maternal communication and in mammals, this dialogue is defined as the maternal recognition of pregnancy (MRP) (Thatcher et al., 1986). The MRP is related to sustaining the functional lifespan of the corpus luteum (CL), and in ruminants, the main signal is identified as Interferon-Tau (IFNT), a protein synthesized and secreted by the trophectoderm of the embryo, between days 10 and 21-25 (Roberts, 1996); recent studies identified IFNT effects around Day 7 in the uterus (Sponchiado et al., 2017). The major function of IFNT has been determined to inhibit the luteolytic mechanisms to maintain progesterone (P4) secretion by the CL (Gray et al., 2006; Spencer et al., 2007).

The IFNT signals use the IFNAR1 (α-subunit) and IFNAR2 (β-subunit) receptors (David, 2002), and this interaction in endometrial cells results in the activation of canonical signaling pathways (IFN-pathway), leading to the expression of ISGs, the Interferon- stimulated genes (Stewart et al., 2001b; Majoros et al., 2017). These ISGs have roles in implantation, endometrial cells differentiation, stimulating hyperplasia of the endometrial glands, development of the uterine vasculature, and modulation of uterine innate immunity (Bazer, 1989; Spencer et al., 2004b). The action of IFNT on the endometrium is essential for maintaining the production of P4 by the CL (Bazer et al., 2012).

The P4 is the main pregnancy hormone. It is known that its effects on the elongation of the bovine embryo are mediated by changes in endometrial function rather than by direct effects of P4 on the embryo (Clemente et al., 2011), and treatment with P4 early after ovulation increases trophoblast elongation and IFNT secretion (Garrett et al., 1988; O'Hara et al., 2014). Thus, P4 increases embryo growth by acting on the endometrium inducing the secretion of IFNs, and stimulating the production of a variety of embryonic factors, such as protein accumulation within the uterine lumen to support conceptus growth and attachment (Geisert et al., 1992), development of the uterine vasculature, stimulate uterine stromal remodeling, and hyperplasia of the endometrial glands (Bazer et al., 2012).

The optimal concentration of P4 for establishment of pregnancy varies from 2.0 to 5.0 ng/mL (Niemann et al., 1985), consequently, the low circulating P4 during early pregnancy has been associated with reduced pregnancy per insemination (P/AI) (Mann et al., 1999). One strategy to increase P4 concentration is the administration of Human Chorionic Gonadotrophin (hCG) during early luteal phase, to induce a functional accessory corpus luteum (aCL) derived from the first wave dominant follicle after ovulation; these cows that develop an aCL have greater blood perfusion in the primary CL (Voelz et al., 2015). Administration of hCG from five to seven days after estrus or artificial insemination (AI) increases P4 concentrations in lactating dairy cows (Stevenson and Pulley, 2012), beef cows (Nishigai et al., 2002), and beef heifers (Funston et al., 2005); also, cows treated with hCG show greater daily CL growth (1.32 ± 1.63 cm^2^/day) compared to untreated cows, with more P4 production (Wecker et al., 2012). In cows, hCG binds directly to LH receptors acting with a luteotropic effect in CL (De Rensis et al., 2010), and significantly promote CL development (Hazano et al., 2020). These receptors were found also in the bovine endometrium (Freidman et al., 1995).

The endometrium is essential for the establishment and maintenance of pregnancy, through uterine receptivity to implantation and adequate endometrial response to IFNT production (Forde et al., 2012). Thus, it was hypothesized that the increase in circulating P4, by hCG-induced aCL, 5 days after TAI, positively modulates the expression of ISGs in the endometrial cells of pregnant cows, modulating the receptivity of endometrium during the pre-attachment period of pregnancy. The objective of this study was to evaluate IFN-pathway genes (IFNAR2, JAK1, STAT1, STAT2, and IRF9) and interferon-stimulated genes (ISG15, MX1, MX2, and OAS1) expression in endometrium during peri-implantation and early pregnancy in cows treated with hCG. Additionally, the possible relationship between ISGs mRNA expression and maternal blood concentration of P4 was also investigated.

## Methods

### Animals and experimental design

The present study was approved by the Ethics Committee on the Use of Animals of the Federal University of Santa Maria (CEUA-UFSM # 4458161221). Non-lactating cross-bred beef cows (n = 29), in the western region of Rio Grande do Sul/Brazil (29º 35' 21” S, 55º 28' 58” W) were used in the study. The crossbred Brangus and Braford cows were 3 to 6 years old, with body condition score greater than 4.5 (1 = thin and 5 = very fat on a scale from 1 to 5 (Edmonson et al., 1989), absent of any detectable reproductive and clinical disorders during the study period. All animals were kept under the same nutritional conditions, managed under native pasture, with water and mineral salt *ad libitum*.

All cows were synchronized with intramuscular administration (im) of 5.5mg of 17β-Estradiol and 50mg of P4 (Betaproginn®, Boehringer Ingelheim, Brazil) and insertion of the intravaginal device containing 0.96g of P4 (Progestar®, Boehringer Ingelheim, Brazil) in the first day of protocol (D-9). The device remained for 7 days and on its removal (D-2), 1mg of estradiol cypionate and 0.5mg of sodic cloprostenol was administered (im). On Day 0 (D0), fixed-time artificial insemination (FTAI) was performed. All cows were inseminated with commercial semen from the same bull (Braford, ABS Pecplan). At the moment of FTAI, on D0, cows were randomly assigned to the experimental groups: The control group (C; n=13) and hCG group (hCG; n=16). Five days after FTAI (D5), the cows of hCG received a single dose of 2500IU of human Chorionic Gonadotrophin (Chorulon®, MSD Animal Health, im), as previous studies (Beltran and Vasconcelos, 2008). Blood collections and ultrasound evaluations were performed on Days 5 and 14 (D5 and D14) of the experiment. The cows were slaughtered on Day 18 (D18) after FTAI, and the reproductive tracts were collected and immediately placed on ice.

### Endometrial tissue sample collection and conceptus recovery

The cows were slaughtered at a slaughterhouse, and after the collection of reproductive tracts, those tracts were transported to the laboratory in refrigerated thermal boxes containing ice at a temperature of approximately 4 ºC. The tracts were placed in their anatomical position, both ovaries were removed, and the uterus were dissected to remove connective tissues and ligaments. Following the dissection and cleaning of the uterus, uterine flushing was performed to recover the conceptus. The uterus were flushed with 20 mL Dulbeccos’s phosphate-buffered saline (DPBS; pH 7.2). For this flush, the end of the uterine horns ipsilateral and contralateral to the side of ovulation near the uterotubal junction was opened by dissection, the syringes were attached, and flushing fluid was propelled by massage and harvested through the opening of the cervix in a petri-dish (Falcon® Integrid^TM^ dishes, Corning, NY, USA). A hemostatic clamp was placed near the uterotubal junction and fluid was flushed into the uterine horns at the same time and massaged through the uterus. From the uterine flushing for the recovery of the conceptus, the Cows were identified as pregnant (P; presence of conceptus) or non-pregnant (NP; absence of conceptus). Three washes were performed in an attempt to recover the conceptus. After recovering the uterine flushing, they were evaluated on dishes under a stereo microscope for conceptus identify. Intercaruncular endometrial tissues were collected from the uterine horn ipsilateral to the CL as described (Sponchiado et al., 2017). These fragments were snap-frozen in liquid nitrogen and stored at -80 °C for isolation of RNA for RT qPCR.

### Real-time polymerase chain reaction

#### RNA extraction

Approximately 100 mg of endometrial samples were used to extract RNA (TRIzol; Invitrogen, Carlsbad, CA, USA), according to the manufacturer’s recommendations. The NanoDrop-1000 spectrophotometer (Thermo Scientific, USA) was used to measure the RNA concentration and determine its quality. Sample absorbance was measured after excitation with 260- and 280-nm wave length. The RNA concentration mean was 936.39 ng/μl, minimum 259.9 ng/μl, maximum 1.160 ng/μl, and the ratio of absorbance at 260/280 was ~2 (1.99-2.05) for all samples. The RNA was stored at -80 °C until further use for cDNA-synthesis and RT qPCR.

#### Complementary DNA (cDNA) synthesis

Total RNA was diluted with nuclease-free water for a final concentration, and 400ng of total RNA was reverse transcribed into cDNA in 10 μL reaction volume. The cDNA was synthesized using DNAse Amplification Grade (Thermo Fisher, Waltham, MA, USA) for 15 min at 37 ºC to degrade any DNA molecules. The DNAse was inactivated with 1 μL EDTA for 10 min at 65 ºC. Reverse transcription was performed using iScript cDNA synthesis Kit (BioRad, Hercules, CA, USA) for 5 min at 25 ºC followed by 30 min at 42 ºC and 5 min at 85 ºC. The cDNA was prepared for each biological replicate and stored at −20 ºC.

#### Real time PCR gene expression

Quantitative Polymerase Chain Reaction (qPCR) was conducted in a thermocycler (BioRad, Hercules, CA, USA) using cDNA, forward and reverse bovine specific primers, and SYBR fluorophore GoTaq1 Green Master Mix (Promega Corporation, Madison, USA), to study relative mRNA expression of IFNAR2, JAK1, STAT1, STAT2, IRF9, ISG15, MX1, MX2, and OAS1. The final reaction volume of the reaction was 10 μL: 2 μL of cDNA and 8 μL of MIX (5 μL of SYBR, 1 μL of primer forward, 1 μL of primer reverse, and 1 μL of nuclease-free water). To optimize the qPCR assay and to assess the quality of the template, serial dilutions of cDNA templates were used to generate a standard curve, and efficiency between 90 and 110% was considered. Samples were run in duplicate, negative controls for the template were also included, and the results of expression of all analyzed genes were expressed by ΔΔCq method, having ACTB, RPS18, and RPL19 as reference genes. The primers used in the experiment were chosen based on the IFNT pathway and ISGs expression, and the sequences were taken from previous studies from our group and the literature, as shown in Table 1.

**Table 1:** Primers designed for quantitative real-time PCR analysis.

**Gene**	**GenBank**	**Primer Sequence**	**Reference**
**IFNAR2**	NM_174553.2	F: AGCCAGAATGTGTCAGCGAT	Amaral et al. (2021)
		R: AGAACAGGCGCAACATACGA	
**IRF9**	NM_001024506.1	F: GGTTCCTGAGATCGGCCACA	Amaral et al. (2021)
		R: CCTGATTGAGCGGGGACAGT	
**ISG15**	NM_174366.1	F: GGTATCCGAGCTGAAGCAGTT	Amaral et al. (2021)
		R: ACCTCCCTGCTGTCAAGGT	
**JAK1**	XM_024989564.1	F: GGGGTTAGCCGCTTAGGGAG	Amaral et al. (2021)
		R: CCATTCAGAGCTGAGCACTTCC	
**MX1**	NM_173940.2	F: GTACGAGCCGAGTTCTCCAA	Amaral et al. (2021)
		R: ATGTCCACAGCAGGCTCTTC	
**MX2**	NM_173941.2	F: CTTCAGAGACGCCTCAGTCG	Amaral et al. (2021)
		R: TGAAGCAGCCAGGAATAGT	
**OAS1**	NM_001029846.2	F: TAGCCTGGAACATCAGGTC	Amaral et al. (2021)
		R: TTTGGTCTGGCTGGATTACC	
**STAT1**	NM_001077900.1	F: CAGCCCGTTTCAGGATCAGC	Amaral et al. (2021)
		R: CAGTGCAGCTTTCTGCCAGT	
**STAT2**	NM_001205689.1	F: CAAAGGAAGCCCCAGAGCCTA	Amaral et al. (2021)
		R: ACATGCCACTCTTCTGTGTTCA	
**ACTB**	NM_173979.3	F: GGATGAGGCTCAGAGCAAGAGA	Bettegowda et al. (2006)
		R: TCGTCCCAGTTGGTGACGAT	
**RPL19**	NM_000981.4	F: GAAAGGCAGGCATATGGGTA	Forde et al. (2013)
		R: TCATCCTCCTCATCCAGGTT	
**RPS18**	NM_001033614.2	F: TGGAGAGTATTGCGCCTTCTC	Sá et al. (2017)
		R: CACAAGTTCCACCACACTATTGG	

### Blood sampling and P4 concentration

Blood samples were collected from the coccygeal vein using a 21G needle coupled to a vacuum collection system (BD Vacutainer1) into 4 mL tubes with a clot activator. The collections were performed at the time of FTAI (Day 0) and on Days 5 and 14 following AI. Blood was obtained in two tubes of 4 mL for each experimental time point and for the determination of blood concentration of P4. After collection, blood was centrifuged at 1800 X g for 15 min. Serum was stored at -20 °C until the assay. The concentration of P4 was determined in serum by chemiluminescent assay kit (ADVIA Centaur, Siemens). Samples were run in duplicate and were analyzed at the same plate. The assay sensitivity was 0.05 ng/mL, and the intra-assay coefficient of variation was 2.0% for P4.

### Ultrasound examinations

Transrectal ultrasound examinations of the ovaries was performed with a Mindray device, model dp10, equipped with a linear transducer and 5.0/7.5 Mhz wave emission, with a total gain of 70%. The images were performed in B-mode to determine the positions and measurement of the preovulatory follicle (POF) and CL structures. The ultrasound POF assessment was performed before insemination (D0) and that of the CL, five and 14 days after TAI (D5 and D14). The objective of the POF evaluation was to ensure that all cows in the experiment had similar ovarian characteristics, discarding the influence of POF size on CL size during the analyzes. The transversal distances perpendicular to each other were measured and from their average, the diameter of the FOP and CL was calculated. The other examinations were carried out on Days 5 and 14 to evaluate the size of the corpus luteum after ovulation and the presence of an accessory corpus luteum (aCL), respectively.

### Statistical analysis and graphical illustration

All data analysis was performed using the JMP14 Software (SAS Institute Inc., Cary, NC, USA). The continuous data were checked for normality using Shapiro-wilk test. Due to the not-normally distributed residuals, data of all target genes in the final models were log-transformed (natural logarithm). The concentration of P4 and mRNA abundance was analyzed by ANOVA followed by Student’s t test. Results are presented as mean ± standard error of the mean (SEM) and are considered different at P<0.05.

## Result

### Ovarian characteristics and CL parameters

The corpus luteum was evaluated on Days 5, 14 and 18 after FTAI. On Days 5 and 14, the CL was evaluated by ultrasonography for the diameter assessment; on Day 18, during the slaughter, the CL was assessed for volume, weight, and total luteal mass. The POF size on Day 0, CL diameter on Day 5 and 14, and volume and weight on Day 18 did not differ between groups. However, the hCG group had greater total luteal mass on Day 18 compared to C group. For total luteal mass, the weight of the primary CL plus the weight of aCL was considered. The data are shown in Table 2.

**Table 2:** Ovarian characteristics on Days 0, 5 and 14, and CL parameters on Day 18 of pregnancy in cows. The data are presented with Mean ± SEM.

**Variable**	**Groups**		
	**C**	**hCG**	**p-value**
Size of POF Day 0	12.59 ± 0.57^a^	12.17 ± 0.50^a^	0.54
Size of CL Day 5 (mm)	18.88 ± 0.60^a^	18.71 ± 0.52^a^	0.96
Size of CL Day 14 (mm)	22.60 ± 1.11^a^	22.83 ± 0.97^a^	0.98
Volume CL Day 18 (cm^3^)	2.83 ± 0.43^a^	3.07 ± 0.36^a^	0.91
Weight CL Day 18 (g)	3.59 ± 0.40^a^	3.46 ± 0.34^a^	0.86
Total Luteal Mass Day 18 (g)	3.48 ± 0.68^a^	6.37 ± 0.56^b^	0.004

Different letters represent significance at P<0.05 between C and hCG groups of cows.

### Conceptus recovery rate, aCL induction rate, and P4 concentration

There was no difference between the conceptus recovery rate of the cows in C (53.84% - 7/13) compared to the hCG (37.5% - 6/16). The cows in the hCG had a 62.5% aCL formation rate (10/16), and none of the cows in C developed aCL (0/13). The administration of the hCG resulted in greater serum P4 concentration in the hCG compared to the C on D14 (14.57 ± 1.09ng/ml and 22.36 ± 1.38ng/ml, C and hCG group, respectively). These results are shown in Table 3.

**Table 3:** Effect of hCG on embryo recovery rate, accessory corpus luteum formation rate, and concentration of progesterone (P4) on days 5 and 14 of experimental design.

**Variable**	**Groups**		
	**C**	**hCG**	**p-value**
Conceptus Recovery rate	53.84%ª (7/13)	37.5%ª (6/16)	0.82
aCL formation rate	- ª (0/13)	62.5%^b^ (10/16)	0.0024
P4 D5 (ng/ml)	4.46 ± 0.48^a^	4.87 ± 0.42^a^	0.52
P4 D14 (ng/ml)	14.04 ± 1.09^a^	22.36 ± 1.38^b^	<.0001

Different letters represent significance at P<0.05 between C and hCG groups of cows.

### The mRNA expression of IFN-pathway genes in cows on Day 18 post-FTAI

**Figure 1:**
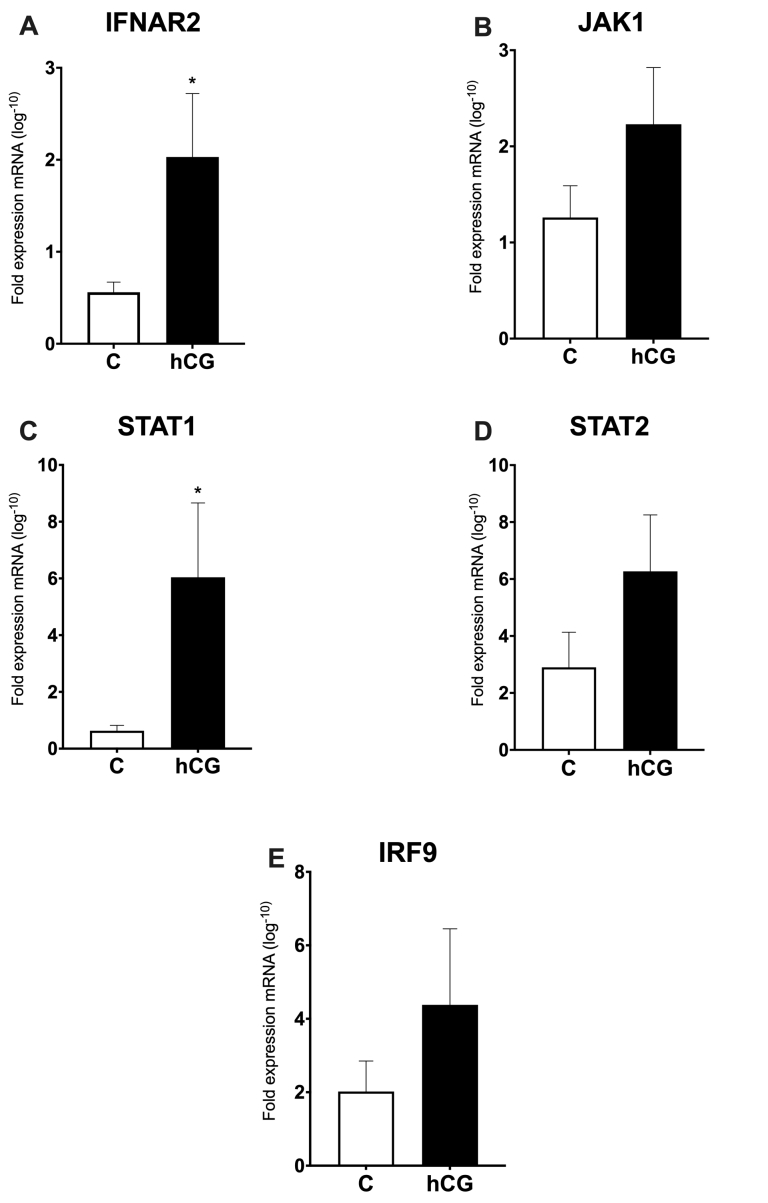
The mRNA expression of IFN-pathway genes in cows on Day 18 post FTAI. qPCR of IFN-pathway genes (A: IFNAR2, B: JAK1, C: STAT1, D: STAT2, and E: IRF9) in bovine endometrial tissues on cows with absence of hCG (C) and cows treated with a single dose of 2.500IU of hCG post FTAI (hCG). Relative expression values are shown (mean ± SEM) on endometrial in Pregnant Control group cows (white bars) and Pregnant hCG group (black bars). Differences in gene expression between treatments (P<0.05) are indicated by asterisk (*).

The IFNT-pathway had mRNA expression of IFNAR2, JAK1, STAT1, STAT2, and IRF9 evaluated in the bovine endometrium, after the use of the hCG, on endometrial ipsilateral portion, on Day 18 after FTAI. Only cows whose embryos were recovered were used. The analyzes were composed of 7 pregnant cows in the Control group and 6 cows in the hCG group. The target genes selected as representatives for the IFNT-pathway were regulated in this study. The results are shown in Figure 1. Of the evaluated genes, only the IFNAR2 (Figure 1A) and STAT1 mRNA abundance (Figure 1C) were upregulated (P<0.05) on cows of the hCG group compared to the C group. The other genes (Figures 1B, 1D, 1E) were not affected by hCG treatment, showing similar expression of mRNA between groups.

### The mRNA abundance of ISG15, MX1, MX2, and OAS1 in cows on Day 18 post FTAI

**Figure 2:**
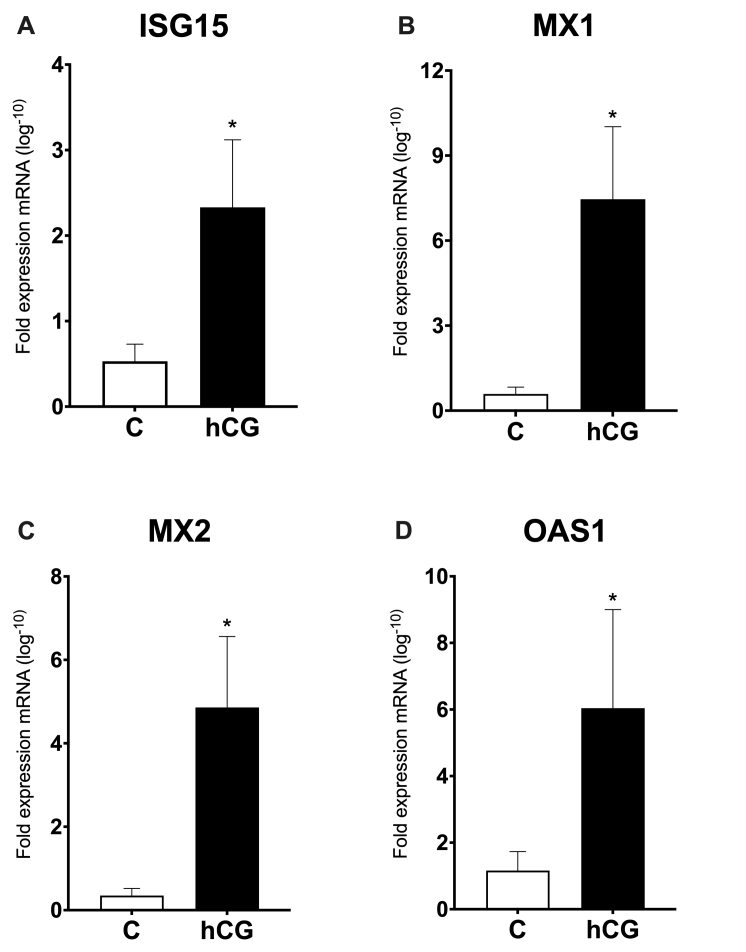
The mRNA expression of ISGs in cows on Day 18 post FTAI. Quantitative real-time PCR analysis of four ISGs genes (A: ISG15, B: MX1, C: MX2, and D: OAS1) in bovine endometrial tissues on cows with absence of hCG (C) and cows treated with a single dose of 2.500IU of hCG post FTAI (hCG). Relative expression values are shown (mean ± SEM) on endometrial in Pregnant Control group cows (white bars) and Pregnant hCG group (black bars). Differences in gene expression between treatments (P< 0.05) are indicated by asterisk (*).

The mRNA abundance of ISG15, MX1, MX2, and OAS1 was evaluated in the bovine endometrium, on endometrial ipsilateral portion, on Day 18 after FTAI. Only cows whose embryos were recovered were used. The analyzes were composed of 7 pregnant cows in the Control group and 6 cows in the hCG group. The results are shown in Figure 2. The mRNA for ISG15, MX1, MX2, and OAS1 were upregulated on pregnant cows for hCG group, compared to C group.

## Discussion

Understanding the impact of a viable conceptus on the maternal uterus may contribute to the development of new technologies for a more efficient and successful pregnancy, benefiting livestock production. This study describes the impact of the use of hCG five days after FTAI, on mRNA of IFNT-pathway and Interferon-Stimulated Genes (ISGs) on the endometrium of cows on Day 18 of pregnancy. The results present that the administration of hCG, through increased the serum P4 concentration, increases IFN-pathway and ISGs mRNA abundance in bovine endometrium at Day 18 of pregnancy.

The IFNT, a glycoprotein secreted by embryonic trophectoderm is the main component of the maternal recognition of pregnancy mechanism, is a member of the type I family of interferons, and acts through a common receptor, IFNAR, which consists of two polypeptide subunits, IFNAR1 and IFNAR2 (Roberts et al., 1997). The IFNAR2 has an extracellular region and is the subunit that contributes most to the interferon binding (Lim and Langer, 1993). It is known that IFNAR2 is more involved in directing normal uterine preparation for bovine embryo implantation and plays a major role in establishing the dialog between the late blastocyst and the uterus (Wang et al., 2022). In the present study, IFNAR2 mRNA abundance was upregulated in hCG group, suggesting that the hCG can increase uterine receptivity during pregnancy, probably through increased P4, and provides greater binding of IFNT to its receptor, stimulating a greater biological response; this greater biological response can be understood as the greater stimulation of ISGs on the endometrium.

The IFNT-pathway, and STAT proteins represent a family of transcription factors involved in cell proliferation, differentiation, and apoptosis (Ivashkiv and Hu, 2004), and the positive effects of IFNT are mediated primarily by these proteins (Stewart et al., 2001b), and only STAT1 and STAT2 remain persistently phosphorylated upon long-term stimulation of cells with IFNT (Stewart et al., 2001a). It is known that STAT1 transcript level is greater in the bovine pregnant endometrium, its expression significantly increases from Day 16 to Day 20, and treatments of exogenous P4 did not affect STAT1 transcript and protein levels in cows (Vitorino Carvalho et al., 2016), suggesting no impact of P4 on regulation of STAT1 mRNA abundance in the endometrium of cows. In the present study, pregnant cows of hCG group had an increase of STAT1 mRNA, and these results lead us to suggest that there may exist a direct modulation by hCG of the STAT1 mRNA abundance, independent of the high concentration of P4 in these cows. Moreover, the increase only in STAT1 but not STAT2 mRNA in the present study can be explained by the fact that STAT1’s ability to homodimerize in IFNT-stimulated cells on the endometrium and bound to gene promoters independent of STAT2 (Seegert et al., 1994). Other studies suggest an interaction between the recruitment of STAT1 and the transcriptional induction of IFNT early target genes by IFNT (Vitorino Carvalho et al., 2016). In our results, there is also an upregulation of STAT1 together with the ISGs, like ISG15, MX1, MX2, and OAS1 mRNA. We believe that this greater increase of STAT1 mRNA abundance was stimulated by IFNT; that this production of IFNT is the result of an indirect action of hCG in the conceptus through direct stimulation on the endometrium through increasing circulating P4. Embryonic quality analysis, as well as measurement of IFNT are part of ongoing studies.

Whereas STAT1 mRNA was increased in the hCG group, STAT2, and IRF9 mRNA showed no difference in the hCG group. The IRF9 binds to STAT2 in IFNT-stimulated cells and this complex (STAT2/IRF9) has been described to be sufficient to activate the IFNT-stimulated response element (ISRE) and finally stimulated the ISGs (Kraus et al., 2003). It explains why the expression of STAT2 and IRF9 genes behave similarly in the present study in both groups. Indeed, specific modulation of STAT1 activity has appeared as a potential mechanism to drive STAT2/IRF9-dependent; thus, our result suggests that hCG can induce a STAT1-dependent and STAT2-IRF9 independent response to the stimulation of IFNT; or even, IFNT can use another signaling pathway to induce ISGs.

Several proteins outside the basic pathway machinery could influence the JAK/STAT signaling and are regulated by intrinsic and environmental stimuli (Cheng et al., 2019), which may explain the fact that there was no difference of JAK1 mRNA by treatment with hCG. Among all these stimuli for the JAK-STAT pathway, the activation by IFNT induces the expression of classical ISGs, including ISG15; for this reason, we analyzed whether hCG also induced overexpression of ISGs in treated cows.

The ISGs have a specific function in conceptus-endometrial interactions have been associated with cellular antiviral responses (Pestka 2007), and in the present study, the hCG increased abundance of classic ISGs such as ISG15, MX1, MX2, and OAS1 mRNA on the endometrium at 18 days of pregnancy. The majority of ISGs are induced or increased in response to the conceptus or expressed in the endometrial epithelium allowing direct regulation by P4 through P4 receptor (PR) activation. However, continuous signaling of the endometrium to P4 downregulates PR expression (Spencer et al., 2004a); We can suggest that hCG minimizes these effects of PR self-regulation, providing greater expression of ISGs.

Among the analyzed genes, ISG15 mRNA is the most upregulated ISG in the pregnant endometrium, it is expressed in endometrial epithelial cells with maximum abundance of mRNA around Day 18 in pregnant cows (Austin et al., 1996), and this is related to several activities such as gene transcription, signal transduction, and cell cycle (Forde et al., 2013). The MX2 mRNA was upregulated in the pregnant cows of the hCG group. The MX2 belongs to a large GTPase family and is recognized as an intracellular antiviral protein (Mansouri-Attia et al., 2009), and known that embryonic losses later in bovine pregnancy were associated with lower fold increases in MX2 mRNA abundance (Stevenson et al., 2007). Likewise, in other species, the MX1 functions outside the immune response to viral infection during early pregnancy, perhaps in the processes of endometrial secretion or uterine remodeling accompanying pregnancy recognition (Horisberger, 1992). The OAS1 may play roles in the control of apoptosis, cellular growth, and differentiation (Salzberg et al., 1997), and the fact the cows of the hCG group had more expression of OAS1 mRNA, is yet another indication that leads us to believe that hCG can act indirectly on the conceptus, increasing the IFNT production and embryo development. These data, together with our study, allow us to suggest that hCG may play an important role in modulating uterine receptivity, by increasing circulating P4, and even in modulating the maternal immune system when it induces greater expression of these genes in the maternal endometrium on Day 18 of pregnancy.

**Figure 3:**
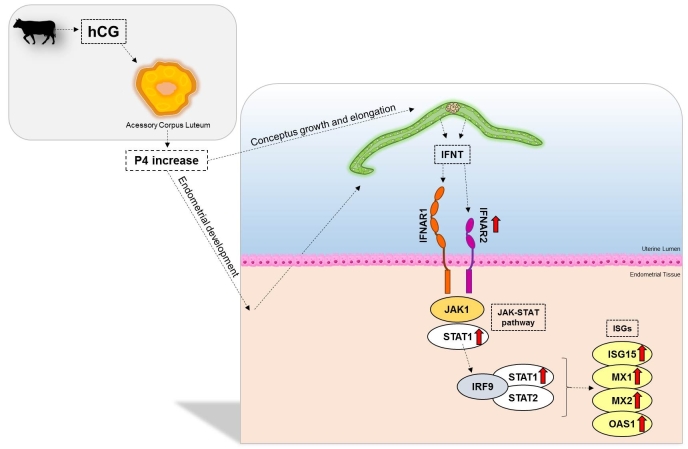
Proposal for the action of hCG on the bovine endometrium at 18 days of pregnancy. The figure represents the proposed action of hCG on the bovine endometrium and conceptus at 18 days of pregnancy. The hCG administered 5 days after FTAI causes ovulation of a dominant follicle and formation of an accessory corpus luteum. This accessory corpus luteum increases circulating progesterone concentrations, resulting in possible changes in the uterine environment, providing greater stimulus for embryonic development and elongation. A larger conceptus secretes more IFNT, greater IFNAR2 expression contributes to greater IFNT binding, with more STAT1 stimulation, increasing the expression of ISGs, and improving signaling of maternal recognition of pregnancy. The red arrows indicate genes that were upregulated after hCG treatment.

Thus, the hCG administered 5 days after FTAI causes ovulation of a dominant follicle and formation of an accessory corpus luteum. This accessory corpus luteum increases circulating P4 concentrations, resulting in possible changes in the uterine environment, providing greater stimulus for embryonic development and elongation. A larger conceptus secretes more IFNT, greater IFNAR2 expression contributes to greater IFNT binding, with more STAT1 stimulation, increasing the expression of ISGs, and improving signaling of maternal recognition of pregnancy (Figure 3).

## Conclusion

In conclusion, our study presents that hCG increases P4 concentration and upregulates genes of IFNT-pathway signaling and the ISGs on endometrium on Day 18 during early pregnancy. It can play an important role in MPR by IFNT. hCG action could be directly on the corpus luteum, increasing the P4, thus regulating the receptivity of the endometrium, inducing an indirect action on the conceptus, stimulating greater production of IFNT, and contributing to paracrine signaling in the mother uterus.
